# Microbiome–miRNA interactions in the progress from undifferentiated arthritis to rheumatoid arthritis: evidence, hypotheses, and opportunities

**DOI:** 10.1007/s00296-021-04798-3

**Published:** 2021-04-15

**Authors:** Haifeng Lu, Yujun Yao, Jiezuan Yang, Hua Zhang, Lanjuan Li

**Affiliations:** State Key Laboratory for Diagnosis and Treatment of Infectious Disease, National Clinical Research Center for Infectious Diseases, Collaborative Innovation Center for Diagnosis and Treatment of Infectious Diseases, The First Affiliated Hospital, College of Medicine, Zhejiang University, Hangzhou, 310003 Zhejiang People’s Republic of China

**Keywords:** Microbiome, Undifferentiated arthritis, Rheumatoid arthritis, miRNA

## Abstract

The human microbiome has attracted attention for its potential utility in precision medicine. Increasingly, more researchers are recognizing changes in intestinal microbiome can upset the balance between pro- and anti-inflammatory factors of host immune system, potentially contributing to arthritis immunopathogenesis. Patients who develop rheumatoid arthritis from undifferentiated arthritis can face multiple irreversible joint lesions and even deformities. Strategies for identifying undifferentiated arthritis patients who have a tendency to develop rheumatoid arthritis and interventions to prevent rheumatoid arthritis development are urgently needed. Intestinal microbiome dysbiosis and shifts in the miRNA profile affect undifferentiated arthritis progression, and may play an important role in rheumatoid arthritis pathophysiologic process via stimulating inflammatory cytokines and disturbing host and microbial metabolic functions. However, a causal relationship between microbiome–miRNA interactions and rheumatoid arthritis development from undifferentiated arthritis has not been uncovered yet. Changes in the intestinal microbiome and miRNA profiles of undifferentiated arthritis patients with different disease outcomes should be studied together to uncover the role of the intestinal microbiome in rheumatoid arthritis development and to identify potential prognostic indicators of rheumatoid arthritis in undifferentiated arthritis patients. Herein, we discuss the possibility of microbiome–miRNA interactions contributing to rheumatoid arthritis development and describe the gaps in knowledge regarding their influence on undifferentiated arthritis prognosis that should be addressed by future studies.

## Introduction

Rheumatoid Arthritis (RA) is an autoimmune disorder that affects more than just the joints particularly fingers and toes, and causes significant morbidity [1]. RA is frequently progressive, and the current medications can only delay the progress but cure. Undifferentiated arthritis (UA), which defined as patients not fulfilling the 2010 ACR/EULAR RA criteria and who did not have a clinical diagnosis other than RA at baseline, can be self-limiting (i.e., the case can undergo spontaneous remission, self-healing, or remain undifferentiated) or develop into rheumatoid arthritis (RA), ankylosing spondylitis, systemic lupus erythematosus, osteoarthritis (OA), or other diseases [2]. A large-scale 2-year follow-up study on the prognosis of UA patients found that only 4.4% of cases spontaneously attained complete remission, while 60.3% remained undifferentiated, and 29.4% progressed to RA [3]. RA is an autoimmune disorder in which the immune system attacks its own tissues and cells, particularly those of the joints. The progression to RA from UA is a continuous and dynamic process, and the resulting spectrum from health to illness is known as the health–disease continuum [4]. Susceptibility factors and immune monitoring are two main research directions regarding RA prevention and control. Much work has been focused on risk factors for RA development. On the basis of data derived from studying patients with preclinical or early stage RA [5], researchers generally believe that there are two main types of RA susceptibility factors: (1) heritable factors, i.e., RA susceptibility genes such as HLA-DR and HLA-DQ, and (2) environmental factors, such as smoking and lifestyle.

Immune dysbiosis profiles, regardless of findings from early serological examination, recent clinical imaging, synovial fluid examination, or synovium biopsy, have been reported to successfully identify patients presenting with UA who were likely to have their disease progress to RA [4, 6, 7]. When such patients are diagnosed very early in their disease course, timely interventions such as the administration of disease-modifying antirheumatic drugs (DMARDs) can improve their prognosis, shorten their disease course, and reduce their disability risk [8]. Although many studies have undeniably furthered our understanding of the molecular mechanisms behind RA development, neither the shared epitope hypothesis of RA susceptibility nor a clear connection between human gene function and RA pathogenesis [9] was confirmed by research conducted on identical twins. Several factors are involved in the induction of RA among cohorts of patients with UA [10]. Recent work has begun to focus on environmental factors and their interactions with genes, but the specific mechanisms are still unclear.

The human intestinal microbiome has attracted attention for its potential utility in precision medicine. Microbiome–host immune system interactions occur via microbial antigens and metabolites [11]; changes in these interactions can upset the balance between the microbiome and host immune system [12], potentially contributing to RA immunopathogenesis. Recent studies have shown that intestinal microbiota dysbiosis accompanies most diseases, including chronic inflammation and tumors [12], cirrhosis/liver cancer [13–15], chronic kidney disease [16], lung disease [17], and arthritis [18]. Increasingly, more researchers are recognizing the critical roles played by the human microbiome (particularly the intestinal microbiome) in the progress and prognosis of RA [18].

Mountains of studies also showed that alterations in miRNA expression contribute to susceptibility of RA/UA (seen in Table 1). However, while many related studies have focused on comparing differences between RA patients and healthy individuals [18, 19], a few studies have compared the differences among UA patients with different prognoses. Additionally, host miRNA–microbiome axis is considered to play a critical role in host–microbiota interactions, and associated with susceptibility in a wide range of diseases such as colorectal cancer [20] and Alzheimer's disease [21]. In this review, we summarize the recent progress regarding microbiome–miRNA interactions, and their potential associations with RA development, and we discuss the future perspectives of viable biomarkers for RA prevention and targeted UA prognosis manipulation.

**Table 1:** The possible miRNAs which reported to play roles in UA/RA

miRNA	UA/RA	Role	References
miR-361-5p	RA	Enriched in apoptosis, tolerance loss, and Wnt pathways	[51]
miRNA-124a	RA	Inhibition in the proliferation and migration	[52]
miRNA-5196	RA	Predict and monitor anti-TNF-α response	[53]
miRNA-126	RA	Inhibit IL-23R mediated TNF-α or IFN-γ production	[54]
miRNA-506	RA	Inhibit RA fibroblast-like synoviocytes proliferation and induces apoptosis	[55]
miRNA-138	RA	Activate NF-κB signaling and PGRN to promote RA via regulating HDAC4	[56]
miRNA-155	RA	miR-155 overexpression or knockdown performed significantly in the development of RA	[57]
miRNA-449a	RA	Inhibit cell proliferation, migration, and inflammation in rheumatoid arthritis fibroblast-like synoviocytes	[58]
miRNA-613	RA	Inhibit proliferation and invasion and induces apoptosis of rheumatoid arthritis synovial fibroblasts	[59]
miR-193a-3p	RA	Regulate proliferation and apoptosis of MH7A cells through targeting IGFBP5	[60]
miRNA-346 miRNA-214	UA	Down-regulated in ST of undifferentiated peripheral inflammatory arthritis	[61]
miR-642B-5p miR-483-3p miR-371 b-5p	UA	Up-regulated in UA → RA vs. UA → UA patients	[62]
miR-25-3p miR-378d	UA	Down-regulated in UA → RA vs. UA → UA patients	[62]

### The intestinal microbiome drives RA pathologic responses in genetically susceptible hosts

The intestinal microbiome drives RA pathologic responses in genetically susceptible hosts. RA genetic research has identified over 100 RA-related gene loci, such as HLA, PTPN22, and TRAF1-C5, and determined that the main RA susceptibility gene in China is HLA-DRB1 [22], PADI4 in Japan [23], PTPN22 in northern European [24], and ACE I/D allele in Arab [25]. However, these loci explain only about 15% of the difference in RA susceptibility risk among individuals [26]. Pioneering studies in animal models have highlighted the importance of non-host genetic factors (intestinal microbiota), revealing that specific microbes in the intestine drive a pathologic immune response to RA in genetically susceptible hosts, thus providing evidence for the involvement of the intestinal microbiome in the development of inflammatory arthritis [27, 28]. For example, *Lactobacillus* and segmented filamentous bacteria in the intestinal microbiota triggered autoimmune diseases and inflammatory arthritis in sterile healthy K/BxN mice, an RA animal model, by inducing Th17 cells [29]. It is well known that Th17 lineage produces cytokines which involved in the pathogenicity of RA; for example GM-CSF, TNF-α, IFNγ, and most of the interleukins [30]. These cytokines, in turn, drive shifts in the composition of the intestinal microbiota and microbial metabolic outputs [31], and thereby play an important role in the progression of autoimmune disorders in RA patients.

A high *Prevotella copri* abundance in the intestines of individuals who are genetically susceptible to RA can drive a pathologic response toward RA development [32]. Maeda et al. [33] colonized germ-free SKG mice (GF-SKG mice) with fecal samples from RA patients or healthy individuals and found that the SKG mice colonized with RA patient fecal samples (*P. copri*-dominated microbiota; RA-SKG mice) displayed more Th17 cells in their large intestine compared with mice colonized with healthy control fecal samples. Furthermore, severe Th17 cell-dependent arthritis appeared in the RA-SKG mice after their injection with low doses of the fungal component zymosan, whereas there were no signs of arthritis when GF-SKG mice were injected with zymosan. These results indicate that intestinal microbiota dysbiosis dominated by *P. copri* can lead to arthritis. Intestinal *P. copri* may contribute to the development of arthritis via the action of superoxide reductase and adenosine phosphate phosphoryl sulfate reductase, the genes for which have been detected in its genome [33]. These two enzymes can enhance the active oxygen tolerance of bacteria, produce thioredoxin, promote the proliferation and inhibit the apoptosis of fibroblast-like synoviocytes, form pannus, and participate in the RA pathologic process [33].

Another study found that some of the low-abundance microbes in the healthy subject controls group were very abundant in untreated RA patients, such as *Collinsella*, the abundance of which was positively correlated with α-aminoadipic acid and asparagine serum levels and related to IL-17A production [34]. Based on subsequent mouse experiments, the researchers concluded that *Collinsella* can change the intestinal permeability and disease severity of mice with experimental arthritis. Together, these findings confirm that certain intestinal bacteria can drive a pathologic immune response toward RA in the host and increase an individual’s risk of developing RA.

Although a causal relationship between the intestinal microbiome and RA development has not yet been comprehensively depicted, it is now clear that the microbiome–metabolite–immune system axis is involved in RA immunopathogenesis. Intestinal microbes maintain homeostasis with the host immune system via their constituents and metabolites. The regulatory effect of metabolites on host immune cells is a vital component of intestinal microbiome–host immune cell interactions; these can trigger chronic inflammation and autoimmunity, which are involved in RA initiation. For example, the short-chain fatty acids and aromatic amines can regulate immune cells through free fatty acid receptor (FFAR) 2, FFAR3, or G protein-coupled receptors metabolites, and they participate in many host immune pathophysiologic processes [35]. Additionally, indole, which is produced from tryptophan through the metabolism of intestinal microbiota, has anti-inflammatory effects; it can inhibit the proinflammatory cytokine production by macrophages via up-regulating PFKFB3 (the main regulator gene of cellular glycolysis) expression, thus significantly reducing the severity of liver steatosis and inflammation [36]. Furthermore, a small proportion of bile acids synthesized by the human liver enter the colon, where they are metabolically transformed by the intestinal microbiome and can act on multiple host nuclear receptors and G-coupled protein receptors, playing a key role in shaping the host innate immune response [37, 38]. Bacterial bile acid metabolites can regulate the number of colonic RORγt^+^ regulatory T (Treg) cells via the vitamin D receptor, and knocking out the bile acid metabolic pathways of intestinal symbiotic bacteria (for example*, Bacteroides fragilis*) inhibited their ability to induce RORγt^+^ Treg cells in murine colons [39]. Additionally, the secondary bile acids (3-oxo LCA and isoallo LCA) metabolized by the intestinal microbiome can regulate Th17 and Treg cell differentiation [40], and the Th17/Treg cell balance is closely related to RA development and severity [41]. However, there is currently no acceptance of a Th17/Treg-based therapeutic strategy to treat RA in humans.

An imbalance in human intestinal microecology, along with the associated changes to the intestinal microbiota metabolic profile, such as a decrease of specific metabolites and the loss of metabolite diversity, will negatively affect the host immune response. Studies on patients with osteoarthritis found that gut microbiome dysbiosis is involved in bacterial metabolite dysbiosis and joint degeneration [42], and similar phenomena were discovered for RA [43]. Interactions between the intestinal microbiota and immune system have been shown to promote and sustain autoimmune rheumatic diseases [44]. Alterations in the function and metabolites of the intestinal microbiome, especially regarding the immune-related inflammatory complex or miRNA metabolites, can cause local or systemic pathophysiologic responses in the host [45, 46], which supposed to be associated with the onset of RA in susceptible individuals. Therefore, the intestinal microbiome may be the most influential non-heritable inducer of RA outside the joints.

### Intestinal microbiome dysbiosis and abnormal miRNA profiles accompany RA development

Although many factors contribute to RA development, the intestinal microbiome has recently been identified as an important pathogenic factor in RA initiation and progression. The contribution of microbiome dysbiosis to RA immunopathogenesis was first reported comprehensively by Zhang et al. [18], who comprehensively analyzed the structure and function of the intestinal microbiota in RA patients in comparison with that of healthy populations (including immediate relatives and relatives without a blood relationship) using metagenomics. They found that *Haemophilus* sp. was enriched in the oral and intestinal flora of healthy controls, and its abundance in the patient group was inversely proportional to the titer of RA autoimmune antibody; *Lactobacillus salivarius* was enriched in the plaque, saliva, and stool of RA patients, especially those with a highly active condition; and, compared with healthy controls, the abundances of some functional genes in the oral and intestinal microorganisms of RA patients were significantly different (including genes related to the transport and metabolism of iron, sulfur, zinc arginine, and citrulline cyclization, which are associated with RA). These findings suggest that abnormalities in the abundance of these functional genes play an important role in the main pathophysiologic processes of RA. In summary, the intestinal microbiome, as well as products of its co-metabolism with the host, can induce host autoimmune diseases and affect RA development.

Abnormal miRNA profiles play a pivotal role in the pathogenesis of many joint injury diseases [47]. Using RNA-seq technology, 63 miRNAs were found to be differentially expressed in the peripheral blood mononuclear cells of RA patients as compared with healthy controls [48]. Lower miRNA-31 levels were also observed in the synovial tissues of RA patients as compared with controls; synovial tissue miRNA-31 is important for RA-induced synovial cell apoptosis [49]. Decreased expression levels of microRNAs (miR-139-3p, miR-204, miR-760, miR-524-5p, miR-136, miR-548d-3p, miR-214, miR-383, and miR-887) in T cells are also involved in RA immunopathogenesis [50]. Additionally, miR-146a is up-regulated in CD4 + T cells from RA patients. The possible miRNA which reported to be associated with UA/RA are shown in Table 1.

Because of the stability, non-invasiveness, and sensitivity of miRNAs, the abnormal expression of miRNAs might be useful for disease diagnosis [63–65]. The serum levels of miR-16-5p, miR-23-3p, miR125b-5p, miR-126-3p, miRN-146α-5p, and miR-223-3p in RA patients were identified as potential novel biomarkers for predicting and monitoring therapy outcomes to anti-TNFα/DMARD combination therapies [66]. The disease specificity of altered miRNA expression profiles is an advantage for their use in the early diagnosis of many diseases. For example, miRNA profiles can be used to distinguish Kashin–Beck disease from osteoarthritis and RA, diseases with clinical manifestations similar to that of Kashin–Beck disease [47]. The importance of therapeutically targeting miRNA has also been demonstrated in various disease models [67]. Further studies with large samples and cell experiments are needed to confirm the therapeutic efficacy of miRNA targeting.

Studies on the role of microbiome dysbiosis in RA development have almost invariably focused on exploring the interaction network between the microbiome, its metabolites, and host immune and miRNA profiles. However, an etiopathogenic role of specific bacteria cannot be inferred by association alone. Therefore, integrating multi-omics studies on RA immunopathogenesis will be important for elucidating targetable mechanisms in cases of preclinical and established RA.

### Bidirectional regulation between intestinal flora and miRNA

The intestinal microbiome composition varies widely among different people; however, for an individual, the composition of the intestinal microbiome is relatively stable, and the structure of its core communities will not change with temporary changes to diet and lifestyle [68, 69]. Human microbiome research generally focuses on the mechanism of selectively shaping the intestinal microbiota. Notably, the intestinal microbiota not only regulates the transcription of host miRNA, but also affects the post-transcriptional modification of some genes [70], thus inducing a host pathophysiologic response; host miRNA can also shape the composition of the intestinal microbiome and regulate the transcription and expression of intestinal microbial genes. The intestinal microbiome has been shown to affect the emotions, social abilities, and cognitive deficits of aseptic mice by changing the expression of miRNA related to anxiety in the brain area; depression-related behaviors could also be induced in this manner and later resolved by intervention with bacteria [70]. Importantly, such interventions restored the miRNA expression profile to normal, suggesting that the intestinal microbiome can regulate the expression of host extraintestinal miRNA and trigger a pathophysiologic response. Tryptophan-derived metabolites produced by the intestinal microbiota can influence miRNA expression in murine white adipose tissue, which is related to the inflammatory pathology of this tissue [71]. Host miRNA regulation by the intestinal microbiome was also found to affect host growth and development [72].

Notably, miRNA can also shape the intestinal microbiota composition and regulate the activity of intestinal bacterial genes [73]. Because intestinal miRNA produced by the host plays an important role in shaping the intestinal microbiome structure and function and is closely related to human health, miRNA has been proposed as a key molecule with which the host regulates intestinal microbiota [73]. Liu et al. [73] screened and identified miRNA isolated from murine and human feces using NanoString digital spatial profiling technology; it revealed that host extracellular miRNA, secreted by small intestinal epithelial cells and Hopx-positive cells in mice and humans, could selectively enter bacteria (such as *Fusobacterium nucleatum* and *Escherichia coli*) to regulate the transcription and expression of bacterial genes, thus affecting intestinal bacteria growth and shaping the composition of intestinal flora. When these researchers specifically knocked out Dicer, an enzyme responsible for miRNA processing, in murine small intestinal epithelial cells and Hopx-positive cells, fecal miRNA was reduced, and the mice showed symptoms of uncontrolled intestinal bacteria growth and colitis aggravation. Transplantation of the intestinal miRNA from normal mice to these defective mice was able to restore the intestinal microecological balance and improve the physical condition of the animals. Additionally, miRNAs’ regulation of microbial gene expression and growth was also reported in neurodegenerative diseases [74].

**Fig. 1:**
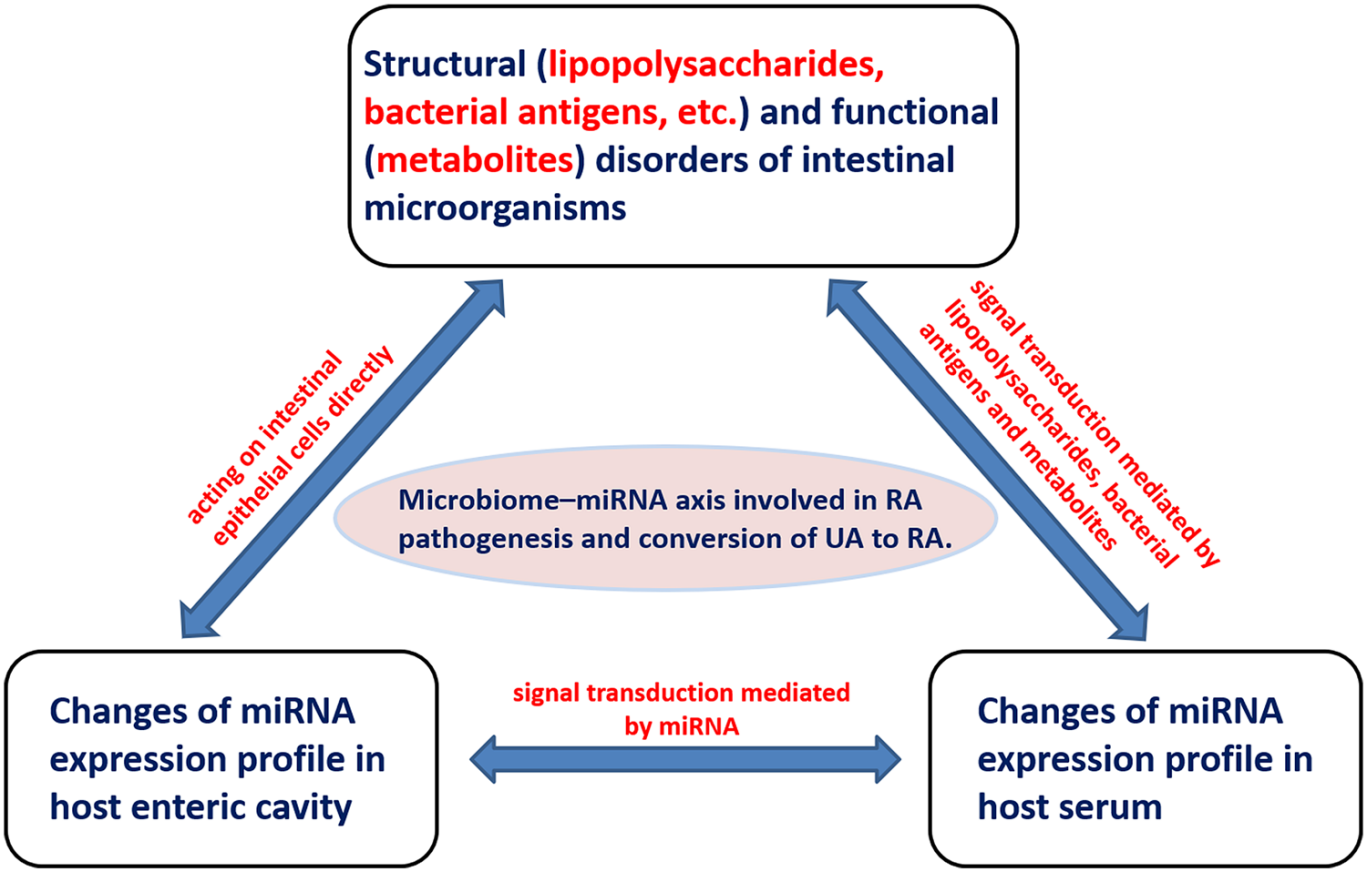
The hypotheses of microbiome–miRNA interactions in the progress from undifferentiated arthritis to rheumatoid arthritis

Host miRNA action provides an important mechanism for maintaining intestinal microbial homeostasis. In addition to the intestinal flora being related to the host extraintestinal immune function, it is also capable of affecting the host extraintestinal miRNA expression, known as the “microbiome–miRNA axis” (Fig. 1). Its roles in the pathophysiology of immune health and diseases were discussed by Li et al. [75], which suggested a promising new approach for presenting valuable diagnostic tools in UA/RA.

Studies on the mechanisms of development for host extraintestinal diseases have found that the intestinal microbiome is related to the host miRNA regulatory network. Abnormal miRNA profile changes are related to a variety of diseases, and their role in arthritis development is clear. Manipulating the intestinal microbiota and miRNA may improve treatment for this disease.

### Predicting RA development in UA patients to prevent RA

Administering DMARDs to patients during the early initiation of arthritic disease is beneficial for relieving disease activity and radiographic joint damage [76]. Thus, it is vital for clinicians to identify patients with UA whose disease will evolve into RA if left untreated and to implement an appropriate treatment strategy. Such patients may exhibit a particular clinical presentation during the process of UA evolving into RA [2]. This theoretical presentation could be used to predict the evolution of RA in UA patient cohorts. However, once UA patients have obvious symptoms of joint damage visible by radiographic examination, it is too late for disease intervention. Consequently, much research has focused on attempting to predict the prognosis of UA and on identifying the early inducing factors of RA [77].

In the preclinical stages of RA, even before synovial biopsy and joint MRI show joint tissue damage, antibody to cyclic citrullinated peptide (anti‐CCP) and rheumatoid factor (RF) are already detectable in the peripheral blood [78]. Furthermore, antibody titer and epitope specificity are increased, and proinflammatory cytokine levels are abnormally high a few months before obvious disease, i.e., synovitis, appears [79]. Together, these findings indicate that patients develop autoimmune disorders before developing joint injuries. Although many studies have tried to describe the pathologic history of UA/RA, the field remains in need of non-invasive, stable, sensitive biomarkers that specifically identify the subgroup of patients with UA who will develop RA.

Multicenter studies with larger cohorts that investigate shifts in the clinical variables of patients with different UA prognoses will be vital to predicting the future development of RA in UA cohorts. Some clinical variables, such as anti‐CCP levels, polyarthritis, symmetric arthritis, and erosions visible on radiographs, have the potential to predict future RA development in UA cohorts. For example, the Leiden prediction rule, which takes into account the tender joint count, duration of morning stiffness, and duration of arthritis, was reported to have a potential role in predicting RA development [80]. However, the presence of anti‐CCP, duration of morning stiffness, number of swollen joints, radiographic progression, modified disease activity score (DAS), and percentage of RF-positive individuals were similar between RA patients who initially presented with UA and those who presented with RA directly [2]. To better prevent RA development and progression, ideal biomarkers should dynamically and specifically reflect the disease pathology [81] and be capable of clearly distinguishing among UA patients who will undergo different disease evolutions.

Many investigations on RA patients have revealed microbiome dysbiosis and abnormal miRNA profiles in these individuals. Emerging evidence suggests a bidirectional regulatory mechanism between the intestinal microbiota and miRNA in patients with UA/RA during the presentation of UA disease, and the intestinal microbiome may affect an individual’s UA prognosis. Additionally, the microbiome and miRNA have the highest specificities and positive predictive values for human health and disease states [82].

## Conclusion

The role of the microbiome and miRNA in the process of UA evolving into RA is an active area of RA research, and the mechanism of interaction is still unclear. Increased understanding of how these two factors interact and of their involvement in disease progression may provide mechanistic insight into RA development and lead to improved treatments for modifying UA and preventing RA. Additionally, we speculate that the miRNA profile as well as the microbiome composition and function differ between the subgroup of UA patients who progress to having RA and those who present directly with RA. Although key alterations in the oral and intestinal microbiomes have been demonstrated in patients who present with RA, the natural microbiome characteristics in patients who present with UA and subsequently develop RA are unknown, as are the shifts that occur during this progression. Therefore, we recommend that additional research be conducted on the abnormal alterations (dysbiosis) in the intestinal microbiome and miRNA of individuals as their UA evolves into RA.

## References

[1] McInnes IB, Schett G (2007). Cytokines in the pathogenesis of rheumatoid arthritis. Nat Rev Immunol.

[2] van Aken J, van Dongen H, le Cessie S, Allaart CF, Breedveld FC, Huizinga TW (2006). Comparison of long term outcome of patients with rheumatoid arthritis presenting with undifferentiated arthritis or with rheumatoid arthritis: an observational cohort study. Ann Rheum Dis.

[3] Ramagli A, Corbacho I, Linhares F, de Abreu P, Teijeiro R, Garau M (2015). Characteristics of patients with early-onset arthritis in Latin America: description of the REPANARC cohort. J Clin Rheumatol.

[4] Chen D, Li H, Liang L, Xiao Y, Xu T, Qiu Q (2013). Clinical features and independent predictors in the further development of rheumatoid arthritis in undifferentiated arthritis. Rheumatol Int.

[5] Gerlag DM, Raza K, van Baarsen LG, Brouwer E, Buckley CD, Burmester GR (2012). EULAR recommendations for terminology and research in individuals at risk of rheumatoid arthritis: report from the Study Group for Risk Factors for Rheumatoid Arthritis. Ann Rheum Dis.

[6] McNally E, Keogh C, Galvin R, Fahey T (2014). Diagnostic accuracy of a clinical prediction rule (CPR) for identifying patients with recent-onset undifferentiated arthritis who are at a high risk of developing rheumatoid arthritis: a systematic review and meta-analysis. Semin Arthritis Rheum.

[7] Arana-Guajardo A, Perez-Barbosa L, Vega-Morales D, Riega-Torres J, Esquivel-Valerio J, Garza-Elizondo M (2014). Application of a prediction model for the progression of rheumatoid arthritis in patients with undifferentiated arthritis. Reumatol Clin.

[8] Brinkmann GH, Norli ES, Kvien TK, Haugen AJ, Grovle L, Nygaard H (2017). Disease characteristics and rheumatoid arthritis development in patients with early undifferentiated arthritis: a 2-year followup study. J Rheumatol.

[9] Gregersen PK, Silver J, Winchester RJ (1987). The shared epitope hypothesis. An approach to understanding the molecular genetics of susceptibility to rheumatoid arthritis. Arthritis Rheum.

[10] Scherer HU, Häupl T, Burmester GR (2020). The etiology of rheumatoid arthritis. J Autoimmun.

[11] Schroeder BO, Backhed F (2016). Signals from the gut microbiota to distant organs in physiology and disease. Nat Med.

[12] Gagliani N, Hu B, Huber S, Elinav E, Flavell RA (2014). The fire within: microbes inflame tumors. Cell.

[13] Qin N, Yang F, Li A, Prifti E, Chen Y, Shao L (2014). Alterations of the human gut microbiome in liver cirrhosis. Nature.

[14] Ren Z, Jiang J, Lu H, Chen X, He Y, Zhang H (2014). Intestinal microbial variation may predict early acute rejection after liver transplantation in rats. Transplantation.

[15] Ren Z, Li A, Jiang J, Zhou L, Yu Z, Lu H (2019). Gut microbiome analysis as a tool towards targeted non-invasive biomarkers for early hepatocellular carcinoma. Gut.

[16] Evenepoel P, Poesen R, Meijers B (2017). The gut-kidney axis. Pediatr Nephrol.

[17] He Y, Wen Q, Yao F, Xu D, Huang Y, Wang J (2017). Gut-lung axis: the microbial contributions and clinical implications. Crit Rev Microbiol.

[18] Zhang X, Zhang D, Jia H, Feng Q, Wang D, Liang D (2015). The oral and gut microbiomes are perturbed in rheumatoid arthritis and partly normalized after treatment. Nat Med.

[19] Picchianti-Diamanti A, Panebianco C, Salemi S, Sorgi ML, Di Rosa R, Tropea A (2018). Analysis of Gut Microbiota in Rheumatoid Arthritis Patients: Disease-Related Dysbiosis and Modifications Induced by Etanercept. Int J Mol Sci.

[20] Dong J, Tai JW, Lu LF (2019). miRNA-Microbiota interaction in gut homeostasis and colorectal cancer. Trends Cancer.

[21] Alexandrov P, Zhai Y, Li W, Lukiw W (2019). Lipopolysaccharide-stimulated, NF-kB-, miRNA-146a- and miRNA-155-mediated molecular-genetic communication between the human gastrointestinal tract microbiome and the brain. Folia Neuropathol.

[22] Okada Y, Wu D, Trynka G, Raj T, Terao C, Ikari K (2014). Genetics of rheumatoid arthritis contributes to biology and drug discovery. Nature.

[23] Suzuki A, Yamada R, Chang X, Tokuhiro S, Sawada T, Suzuki M (2003). Functional haplotypes of PADI4, encoding citrullinating enzyme peptidylarginine deiminase 4, are associated with rheumatoid arthritis. Nat Genet.

[24] Yamamoto K, Okada Y, Suzuki A, Kochi Y (2015). Genetics of rheumatoid arthritis in Asia—present and future. Nat Rev Rheumatol.

[25] Song GG, Bae SC, Kim JH, Lee YH (2015). The angiotensin-converting enzyme insertion/deletion polymorphism and susceptibility to rheumatoid arthritis, vitiligo and psoriasis: a meta-analysis. J Renin Angiotensin Aldosterone Syst.

[26] Lenz TL, Deutsch AJ, Han B, Hu X, Okada Y, Eyre S (2015). Widespread non-additive and interaction effects within HLA loci modulate the risk of autoimmune diseases. Nat Genet.

[27] Abdollahi-Roodsaz S, Joosten LA, Koenders MI, Devesa I, Roelofs MF, Radstake TR (2008). Stimulation of TLR2 and TLR4 differentially skews the balance of T cells in a mouse model of arthritis. J Clin Invest.

[28] Wu HJ, Ivanov II, Darce J, Hattori K, Shima T, Umesaki Y (2010). Gut-residing segmented filamentous bacteria drive autoimmune arthritis via T helper 17 cells. Immunity.

[29] Kugyelka R, Kohl Z, Olasz K, Mikecz K, Rauch TA, Glant TT (2016). Enigma of IL-17 and Th17 cells in rheumatoid arthritis and in autoimmune animal models of arthritis. Mediators Inflamm.

[30] Marwaha AK, Leung NJ, McMurchy AN, Levings MK (2012). TH17 cells in autoimmunity and immunodeficiency: protective or pathogenic?. Front Immunol.

[31] Wang Q, Xu R (2019). Data-driven multiple-level analysis of gut-microbiome-immune-joint interactions in rheumatoid arthritis. BMC Genomics.

[32] Scher JU, Sczesnak A, Longman RS, Segata N, Ubeda C, Bielski C (2013). Expansion of intestinal Prevotella copri correlates with enhanced susceptibility to arthritis. Elife.

[33] Maeda Y, Takeda K (2017). Role of gut microbiota in rheumatoid arthritis. J Clin Med.

[34] Chen J, Wright K, Davis JM, Jeraldo P, Marietta EV, Murray J (2016). An expansion of rare lineage intestinal microbes characterizes rheumatoid arthritis. Genome Med.

[35] Garrett WS (2020). Immune recognition of microbial metabolites. Nat Rev Immunol.

[36] Ma L, Li H, Hu J, Zheng J, Zhou J, Botchlett R (2020). Indole alleviates diet-induced hepatic steatosis and inflammation in a manner involving myeloid cell PFKFB3. Hepatology.

[37] Wang YD, Chen WD, Yu D, Forman BM, Huang W (2011). The G-protein-coupled bile acid receptor, Gpbar1 (TGR5), negatively regulates hepatic inflammatory response through antagonizing nuclear factor kappa light-chain enhancer of activated B cells (NF-kappaB) in mice. Hepatology.

[38] Guo C, Chen WD, Wang YD (2016). TGR5, not only a metabolic regulator. Front Physiol.

[39] Song X, Sun X, Oh SF, Wu M, Zhang Y, Zheng W (2020). Microbial bile acid metabolites modulate gut RORgamma(+) regulatory T cell homeostasis. Nature.

[40] Hang S, Paik D, Yao L, Kim E, Jamma T, Lu J (2019). Bile acid metabolites control TH17 and Treg cell differentiation. Nature.

[41] Dinesh P, Rasool M (2019). Berberine mitigates IL-21/IL-21R mediated autophagic influx in fibroblast-like synoviocytes and regulates Th17/Treg imbalance in rheumatoid arthritis. Apoptosis.

[42] Favazzo LJ, Hendesi H, Villani DA, Soniwala S, Dar QA, Schott EM (2020). The gut microbiome-joint connection: implications in osteoarthritis. Curr Opin Rheumatol.

[43] du Teil EM, Gabarrini G, Harmsen HJM, Westra J, van Winkelhoff AJ, van Dijl JM (2019). Talk to your gut: the oral-gut microbiome axis and its immunomodulatory role in the etiology of rheumatoid arthritis. FEMS Microbiol Rev.

[44] Konig MF (2020). The microbiome in autoimmune rheumatic disease. Best Pract Res Clin Rheumatol.

[45] Alvarez-Curto E, Milligan G (2016). Metabolism meets immunity: The role of free fatty acid receptors in the immune system. Biochem Pharmacol.

[46] Ambrozkiewicz F, Karczmarski J, Kulecka M, Paziewska A, Niemira M, Zeber-Lubecka N (2020). In search for interplay between stool microRNAs, microbiota and short chain fatty acids in Crohn's disease—a preliminary study. BMC Gastroenterol.

[47] Wu W, He A, Wen Y, Xiao X, Hao J, Zhang F (2017). Comparison of microRNA expression profiles of Kashin-Beck disease, osteoarthritis and rheumatoid arthritis. Sci Rep.

[48] Wen J, Liu J, Zhang P, Jiang H, Xin L, Wan L (2020). RNA-seq reveals the circular RNA and miRNA expression profile of peripheral blood mononuclear cells in patients with rheumatoid arthritis. Biosci Rep.

[49] Luo C, Liang JS, Gong J, Zhang HL, Feng ZJ, Yang HT (2018). miRNA-31 over-expression improve synovial cells apoptosis induced by RA. Bratisl Lek Listy.

[50] Lai NS, Yu HC, Tung CH, Huang KY, Huang HB, Lu MC (2017). The role of aberrant expression of T cell miRNAs affected by TNF-alpha in the immunopathogenesis of rheumatoid arthritis. Arthritis Res Ther.

[51] Romo-Garcia MF, Bastian Y, Zapata-Zuniga M, Macias-Segura N, Castillo-Ortiz JD, Lara-Ramirez EE (2019). Identification of putative miRNA biomarkers in early rheumatoid arthritis by genome-wide microarray profiling: a pilot study. Gene.

[52] Meng HY, Chen LQ, Chen LH (2020). The inhibition by human MSCs-derived miRNA-124a overexpression exosomes in the proliferation and migration of rheumatoid arthritis-related fibroblast-like synoviocyte cell. BMC Musculoskelet Disord.

[53] Ciechomska M, Bonek K, Merdas M, Zarecki P, Swierkot J, Gluszko P (2018). Changes in MiRNA-5196 expression as a potential biomarker of anti-TNF-alpha therapy in rheumatoid arthritis and ankylosing spondylitis patients. Arch Immunol Ther Exp (Warsz).

[54] Gao J, Kong R, Zhou X, Ji L, Zhang J, Zhao D (2018). MiRNA-126 expression inhibits IL-23R mediated TNF-alpha or IFN-gamma production in fibroblast-like synoviocytes in a mice model of collagen-induced rheumatoid arthritis. Apoptosis.

[55] Li D, Zhou Q, Hu G, Wang G (2019). MiRNA-506 inhibits rheumatoid arthritis fibroblast-like synoviocytes proliferation and induces apoptosis by targetting TLR4. Biosci Rep.

[56] Shao L, Hou C (2019). miR-138 activates NF-kappaB signaling and PGRN to promote rheumatoid arthritis via regulating HDAC4. Biochem Biophys Res Commun.

[57] Su LC, Huang AF, Jia H, Liu Y, Xu WD (2017). Role of microRNA-155 in rheumatoid arthritis. Int J Rheum Dis.

[58] Cai Y, Jiang C, Zhu J, Xu K, Ren X, Xu L (2019). miR-449a inhibits cell proliferation, migration, and inflammation by regulating high-mobility group box protein 1 and forms a mutual inhibition loop with Yin Yang 1 in rheumatoid arthritis fibroblast-like synoviocytes. Arthritis Res Ther.

[59] Liu L, Zuo Y, Xu Y, Zhang Z, Li Y, Pang J (2019). MiR-613 inhibits proliferation and invasion and induces apoptosis of rheumatoid arthritis synovial fibroblasts by direct down-regulation of DKK1. Cell Mol Biol Lett.

[60] Qu SP, Li GW, Ma H, Xing Q (2019). MicroRNA-193a-3p participates in the progression of rheumatoid arthritis by regulating proliferation and apoptosis of MH7A cells through targeting IGFBP5. Eur Rev Med Pharmacol Sci.

[61] Alivernini S, Tolusso B, Petricca L, Bui L, Di Mario C, Gigante MR (2018). Synovial predictors of differentiation to definite arthritis in patients with seronegative undifferentiated peripheral inflammatory arthritis: microRNA signature, histological, and ultrasound features. Front Med (Lausanne).

[62] Kurowska W, Kuca-Warnawin E, Radzikowska A, Jakubaszek M, Maslinska M, Kwiatkowska B (2018). Monocyte-related biomarkers of rheumatoid arthritis development in undifferentiated arthritis patients—a pilot study. Reumatologia.

[63] Zhang X, Cai H, Zhu M, Qian Y, Lin S, Li X (2020). Circulating microRNAs as biomarkers for severe coronary artery disease. Med (Baltim).

[64] Jin L, Zhang N, Zhang Q, Ding G, Yang Z, Zhang Z (2020). Serum microRNAs as potential new biomarkers for cisplatin resistance in gastric cancer patients. PeerJ.

[65] Roy B, Yoshino Y, Allen L, Prall K, Schell G, Dwivedi Y (2020). Exploiting circulating MicroRNAs as biomarkers in psychiatric disorders. Mol Diagn Ther.

[66] Castro-Villegas C, Perez-Sanchez C, Escudero A, Filipescu I, Verdu M, Ruiz-Limon P (2015). Circulating miRNAs as potential biomarkers of therapy effectiveness in rheumatoid arthritis patients treated with anti-TNFalpha. Arthritis Res Ther.

[67] Li J, Wan Y, Guo Q, Zou L, Zhang J, Fang Y (2010). Altered microRNA expression profile with miR-146a up-regulation in CD4+ T cells from patients with rheumatoid arthritis. Arthritis Res Ther.

[68] Zhernakova A, Kurilshikov A, Bonder MJ, Tigchelaar EF, Schirmer M, Vatanen T (2016). Population-based metagenomics analysis reveals markers for gut microbiome composition and diversity. Science.

[69] Falony G, Joossens M, Vieira-Silva S, Wang J, Darzi Y, Faust K (2016). Population-level analysis of gut microbiome variation. Science.

[70] Hoban AE, Stilling RM, Moloney RD, Shanahan F, Dinan TG (2017). Microbial regulation of microRNA expression in the amygdala and prefrontal cortex. Microbiome.

[71] Virtue AT, McCright SJ, Wright JM, Jimenez MT, Mowel WK, Kotzin JJ (2019). The gut microbiota regulates white adipose tissue inflammation and obesity via a family of microRNAs. Sci Transl Med.

[72] Seth P, Hsieh PN, Jamal S, Wang L, Gygi SP, Jain MK (2019). Regulation of MicroRNA machinery and development by interspecies S-nitrosylation. Cell.

[73] Liu S, da Cunha AP, Rezende RM, Cialic R, Wei Z, Bry L (2016). The host shapes the gut microbiota via fecal microRNA. Cell Host Microbe.

[74] Hewel C, Kaiser J, Wierczeiko A, Linke J, Reinhardt C, Endres K (2019). Common miRNA patterns of Alzheimer's disease and Parkinson's disease and their putative impact on commensal gut microbiota. Front Neurosci.

[75] Li M, Chen WD, Wang YD (2020). The roles of the gut microbiota-miRNA interaction in the host pathophysiology. Mol Med.

[76] Lard LR, Visser H, Speyer I, vander Horst-Bruinsma IE, Zwinderman AH, Breedveld FC (2001). Early versus delayed treatment in patients with recent-onset rheumatoid arthritis: comparison of two cohorts who received different treatment strategies. Am J Med.

[77] Mochan E, Ebell MH (2008). Predicting rheumatoid arthritis risk in adults with undifferentiated arthritis. Am Fam Physician.

[78] van de Sande MG, de Hair MJ, van der Leij C, Klarenbeek PL, Bos WH, Smith MD (2011). Different stages of rheumatoid arthritis: features of the synovium in the preclinical phase. Ann Rheum Dis.

[79] Sokolove J, Bromberg R, Deane KD, Lahey LJ, Derber LA, Chandra PE (2012). Autoantibody epitope spreading in the pre-clinical phase predicts progression to rheumatoid arthritis. PLoS ONE.

[80] Ghosh K, Chatterjee A, Ghosh S, Chakraborty S, Chattopadhyay P, Bhattacharya A (2016). Validation of Leiden score in predicting progression of rheumatoid arthritis in undifferentiated arthritis in Indian population. Ann Med Health Sci Res.

[81] Dwivedi S, Purohit P, Sharma P (2019). MicroRNAs and diseases: promising biomarkers for diagnosis and therapeutics. Indian J Clin Biochem.

[82] Lee J, Banerjee D (2020). Metabolomics and the microbiome as biomarkers in sepsis. Crit Care Clin.

